# The transcriptome of a *Populus* pseudo-backcross identifies genes and pathways co-expressed with monolignol biosynthesis

**DOI:** 10.1186/1753-6561-5-S7-P119

**Published:** 2011-09-13

**Authors:** Evandro Novaes, Carolina Boaventura-Novaes, Alexandre Coelho, Matias Kirst

**Affiliations:** 1 Escola de Agronomia e Engenharia de Alimentos, Universidade Federal de Goiás, Caixa Postal 131, CEP 74690-900, Goiânia, Brazil; 2 Programa de Pós-Graduação em Genética e Melhoramento de Plantas, Universidade Federal de Goiás, Caixa Postal 131, CEP 74690-900, Goiânia, Brazil; 3 School of Forest Resources and Conservation, University of Florida, PO Box 110410, Gainesville, USA

## Background

Lignin is the main impediment for efficient cellulose extraction and ethanol production from woody tissues. Even though, the biosynthesis of the lignin constituents - the monolignols - is well characterized, little is known about the genetic control of the natural variation in lignin content and composition. The few association studies performed to date in forest species only uncover 5-20% of the heritable variation of quantitative traits such as wood composition. The “missing heritability” can be explained in part by the low resolution of these pioneering association studies in forest species. However, as observed in genome-wide association studies with humans, a large proportion of the “missing heritability” is likely to occur due to other factors, such as the abundance of rare alleles observed in forest species and complex epistatic interaction between genetic elements. Causal rare alleles and genetic interactions remain undetectable with current statistical methods. In order to shed light on possible interactions between lignin biosynthesis genes and other pathways, we analyzed the transcriptome of 181 genotypes of a pseudo-backcross family of *Populus*. The analyses allowed identification of genes and pathways that were highly co-expressed with genes involved in the biosynthesis of monolignols. Correlations at the transcript level offer initial evidence of possible interactions between genetic elements for the production of monolignols.

## Methods

A previously published microarray data [1] was utilized. Briefly, this microarray contains a gene expression probe for every gene annotated in the genome of *Populus trichocarpa* (v1.1.). Microarray chips were hybridized with cDNA synthesized from xylem tissue of 181 genotypes from a pseudo-backcross pedigree of *Populus trichocarpa* x *P. deltoides*. This family was also analyzed for lignin content on xylem tissue [2]. Genetic differences in gene expression allowed us to correlate the transcript abundance of 23 previously identified xylem-specific monolignol biosynthetic-genes [3] against the cDNA levels of all annotated genes of *Populus*. Correlation was estimated based on Spearman’s rank method in R. The top correlated genes (r > 0.75, p-value <0.001) were clustered with the 23 monolignol biosynthetic-genes based on a “Modulated Modularity Clustering” method [4]. The genes clustered with the 23 monolignol biosynthetic-genes were GO annotated based on the BlastP best hit against the *Arabidopsis* gene models. A Fisher’s exact test was applied to identify GO terms enriched within these clusters.

## Results

**Figure 1:**
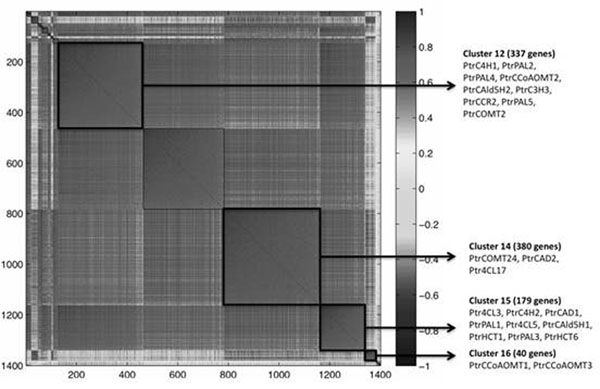
Symmetrical correlation matrix depicting clusters of genes highly co-expressed (r > 0.75) with 23 xylem-specific monolignol biosynthetic-genes. Clusters 12, 14, 15 and 16 contain the 23 monolignol genes, as shown. Genes within these clusters were analyzed for gene ontology term enrichment. Additionally, co-expression networks were built within these four clusters.

Utilizing a correlation threshold of 0.75 (p-value < 0.001), 1369 genes were correlated with at least one of the 23 monolignol biosynthetic-genes. These genes were clustered together with the lignin phenotypic trait and 78 genes that were correlated with lignin (r > 0.45). The 23 monolignol genes were clustered in four groups, containing a total of 936 genes (Figure 1). Surprisingly, the lignin trait itself did not cluster with any other gene, and the vast majority (75%) of the top 78 lignin-correlated genes were grouped on clusters 12 and 15. Clusters 14 and 16 did not have lignin-correlated genes. We analyzed the GO annotation among genes within the four monolignol-related clusters. A Fisher’s exact test was utilized to find GO terms enriched within these clusters when compared to all annotated genes in the genome of *Populus*. Sixty-nine GO terms were significantly enriched (FDR < 0.01) among genes clustered with the 23 monolignol genes: 11 terms are from the ontology Cellular Component (CC), 26 from Molecular Function (MF) and 32 from Biological Process (BP). As expected, most of these MF and BP terms (>65%) are related to lignin, cellulose and hemicellulose biogenesis. This high specificity indicates that even the genes with unknown MF and BP (260 of the 936 monolignol clustered genes) might be involved in cell-wall biogenesis. Enriched GO terms not directly related to cell-wall biogenesis include “drought-recovery”, “salicylic acid catabolism”, “response to cadmium ion” and “response to zinc ion”. In addition to the significantly enriched GO terms, we also constructed a co-expression network including the core, most highly correlated genes (r > 0.85) within each of the four identified, monolignol biosynthetic clusters. These networks, representing the most likely interacting genes, will be presented.

## Conclusion

This work presents a set of approximately 900 genes highly co-expressed with xylem-specific monolignol biosynthesis genes. Many of these genes are currently annotated with unknown function, or are not known to be involved in cell-wall biogenesis. We offer initial evidence towards a role or interaction of these genes in the biosynthesis of lignin and possibly other cell-wall components.
